# The Growth of *Monoraphidium* sp. and *Scenedesmus* sp. Cells in the Presence of Thorium

**DOI:** 10.1100/2012/592721

**Published:** 2012-04-29

**Authors:** Juliana Cristina de Queiroz, Ana Cristina de Melo Ferreira, Antonio Carlos Augusto da Costa

**Affiliations:** ^1^Instituto de Radioproteção e Dosimetria (IRD), SEANA, Avenida Salvador Allende s/n, Jacarepaguá, 22780-160 Rio de Janeiro, RJ, Brazil; ^2^Departamento de Tecnologia de Processos Bioquímicos, Instituto de Química, Universidade do Estado do Rio de Janeiro, Pavilhão Haroldo Lisboa da Cunha, R. São Francisco Xavier 524, Sala 427, 20550-013 Rio de Janeiro, RJ, Brazil

## Abstract

Toxicity of thorium by *Monoraphidium* sp. and *Scenedesmus* sp. was studied. Microalgal cultures were inoculated in ASM-1 medium in presence and absence of thorium. Its effect was monitored by direct counting on Fuchs-Rosenthal chamber and with software. The toxicity of thorium over the species was observed for concentrations over 50.0 mg/L. After 30 days, *Monoraphidium* cells decreased their concentration from 4.23 × 10^6^ to 4.27 × 10^5^ and 8.57 × 10^5^ cells/mL, in the presence of 50.0 and 100.0 mg/L of thorium, respectively. *Scenedesmus* sp. cells were more resistant to thorium: for an initial cell concentration of 7.65 × 10^4^ cells/mL it was observed a change to 5.25 × 10^5^ and 5.12 × 10^5^ cells/mL, in the presence of thorium at 50.0 and 100.0 mg/L, respectively. This is an indication that low concentrations of the radionuclide favored the growth, and that *Scenedesmus* cells are more resistant to thorium than *Monoraphidium* cells. The software used for comparison with direct count method proved to be useful for the improvement of accuracy of the results obtained, a decrease in the uncertainty and allowed recording of the data. The presence of thorium suggests that low concentrations have a positive effect on the growth, due to the presence of the nitrate, indicating its potential for ecotoxicological studies.

## 1. Introduction

Natural radioactive elements, such as uranium and thorium, are widely distributed over the earth crust. However, anthropogenic activities have a marked effect on the natural cycle of these radionuclides, contributing for an outstanding increase in the dispersion of these elements in the environment [1].

The bioaccumulation of toxic substances by microbial cells considerably decreases their concentration in the environment; consequently, their bioavailability decreases. The use of microalgal cells as biomarkers is becoming an efficient tool for the prevention of contamination, considering that biochemical and physiological changes are not observable at organic levels. Bioremediation, on the other side, is being considered as an effective technology for the treatment and removal of contaminants from wastewaters, particularly with a great potential to clean aquatic systems [2]. In this context, Sar et al. [3] studied the uptake capacity of uranium and thorium by soil-isolated *Pseudomonas* cells, for the bioremediation of nuclear wastes. The removal of thorium was also studied by Picardo et al. [4], with the inactivated brown seaweed *Sargassum filipendula *as biosorbent material. In that case, the authors found that the biomass of seaweed was able to recover thorium from solution, however, at very low concentrations, around 1.0 mg/L. This confirms that the lack of biological activity markedly decreases the uptake capacity of the biomass, in comparison to metabolically mediated processes.

According to Barsanti and Gualtieri [5], the estimated number of algal species ranges from one to ten million, most of them microalgal cells. These data emphasize this unexplored field of research, as only a limited number of species has already been studied, from the physiological and biochemical levels [6]. Genera *Monoraphidium* and *Scenedesmus* belong to the order Chlorellales, frequently present as pure culture in plankton [7].

The most traditional way to quantify microalgal population is direct counting, from a known volume of culture, using a counting chamber or hemocitometer—a flat chamber with known area [8, 9]. The observation field is used to quantify the amount of cells present on that space of known volume, being used for all counting chambers. However, factors such as culture density, size, and shape of cells or colonies can markedly affect the choice of a suitable counting chamber.  Table 1 shows some characteristics of commercial cell-counting chambers, regarding cell size and culture density [9].

The direct counting of microbial cells brings some uncertainties associated to this technique, such as effects related to dilution of the sample, operation, and reproducibility. In order to minimize these effects, a computational method for counting cells could contribute to decrease these uncertainties. The use of a suitable software allows the recording of results, preventing uncertainties from dilution of the sample, as it is possible to differentiate between counted and not counted elements, preventing duplicate counts or missing fields in a specific area being investigated.

The objective of the present work was to evaluate the effect of thorium on the growth of green microalgae from the genera *Monoraphidium* and *Scenedesmus*. The specific objectives of the present work included the study of the most suitable pH conditions for the growth of these microalgae, to evaluate the effect of increasing thorium concentrations in the culture medium of the cells, monitored by cell growth, and, finally, to use a software developed by the Institute of Radioprotection and Dosimetry (IRD/CNEN) to be compared with direct counting using counting chambers, with the purpose of decreasing the uncertainties associated to this conventional procedure.

## 2. Materials and Methods

### 2.1. Microalgae and Culture Conditions

The green microalgae *Monoraphidium *sp. (LB-1 strain) and *Scenedesmus *sp. (LB-2 strain) were selected for the present study. These were selected due to the easy cultivation and fast growth. The strains were obtained from the algal bank from the Laboratory of Cyanobacterial Toxicology from CCS/UFRJ, Rio de Janeiro, Brazil.

The culture medium chosen for the experiments was the ASM-1 medium [10]. The medium was autoclaved at 121°C for 20 minutes, followed by the addition of micronutrients solution, in the proportion of 1 mL per liter of culture medium. Aliquots of 5 mL of original microbial cultures were inoculated, under aseptic conditions, in 100 mL ASM-1 medium. The flasks were than closed and kept in a germination chamber (Fanem, Model 347 CDG, Brazil) under 12/12 hour dark-light cycle, at 23°C. The maintenance of the cultures was performed by subculturing the cells every seven days, at 10% v/v. 

### 2.2. Thorium Toxicity Tests

Initially, a test was performed to monitor the growth of the microorganisms, in ASM-1 medium, at pH 6.0 and 8.0. The pH 6.0 and 8.0 were investigated in order to observe the possible occurrence of thorium precipitation in the medium, in the form of hydroxide at high pH values. All thorium toxicity tests were performed with the use of Th(NO_3_)_4_5H_2_O at 1000 ± 23 mg Th/L stock solution. Thorium was then added to the culture medium in the concentrations ranging from 0.5 to 100.0 mg/L, due to previous works demonstrating that concentrations below 0.5 mg/L thorium were easily incorporated by a seaweed biomass [4]. Blank experiments were done without the addition of thorium.

After the addition of thorium to the culture medium, the flasks were autoclaved under the same conditions described, and 0.2 mL of micronutrients solution was added. The inoculated cells constituted 15 mL of the total volume of the flask. Cell growth was monitored through counting the cells during 22 days [11]. Toxic effects of thorium were studied by comparison between the growth curves in presence and absence of the radionuclide. Experiments were performed in triplicate.

### 2.3. Microscopic Counting Using Fuchs-Rosenthal Counting Chamber

The quantification of cells was conducted by direct counting using a Fuchs-Rosenthal counting chamber and an Axioskop 40 Carl-Zeiss microscope. The microscope includes a photographic camera AxioCam coupled to the ocular tube, with high scanning resolution of the observed field. The images obtained were transferred to a computer using the AxioVision software provided.

The Fuchs-Rosenthal chamber is a glass chamber divided in two smaller chambers, 0.2 mm height each one. Each chamber includes 16 large squares (1 mm), and each large square is subdivided into 16 small squares, 250 *μ*m each. Thus, the volume of each square is equal to 0.2 mm^3^. Consequently, 32 large squares can support 0.0064 mL of sample [9]. In order to count the cells, two drops of the sample were placed under each reticulate and the microbial cells inside each of the 16 squares that make each diagonal of the chamber are counted.

In order to decrease the uncertainty of the counting, it was determined that only the elements inside the 16 squares and cells over the triple lines of the upper and left sides of the square would be considered. The number of cells to be counted in each diagonal line must be, at least, 200 cells. If the first count did not reach this number, the next diagonal line would be counted. As a last attempt to reach the minimum number of 200 cells, vertical and horizontal lines were then considered. The amplification used in the microscopy was equal to 20 times the ocular, corresponding to a total increase of 200 fold. For high cell density, the sample must be diluted. The calculation of the cell density, in these cases, was made according to Table 2.

### 2.4. Microscopic Images and Software Development

**Figure 1:**
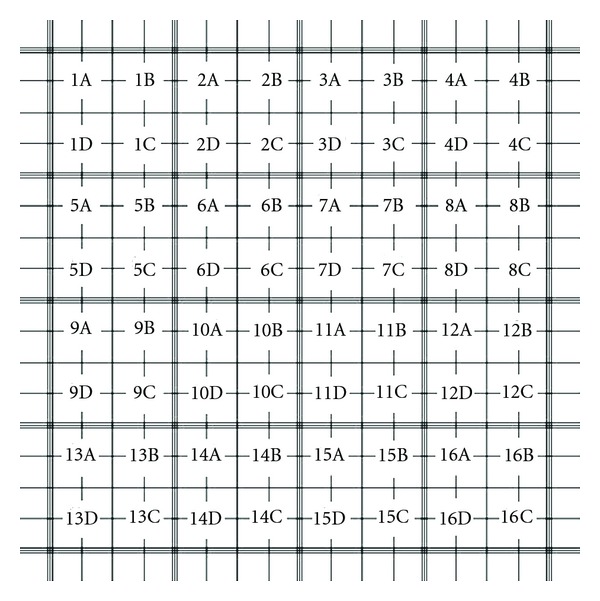
Orientation diagram for the acquisition of images.

After direct counting of the microalgae in the microscope, the images obtained were recorded. The observed field did not correspond to the whole field counted. Just a small number of the small squares appear in the field. The images were then recorded, according to the order presented in Figure 1, from field 1 (A to D) to 16 (A to D). The obtained images were improved using the software Corel Draw X4 (version 14.0.0.567), using the self-contrast function, in order to make it easier the use of the images for the counts using the software. A software was developed by the Institute of Radioprotection and Dosimetry, with the purpose of improving cell counting, for more reliable estimates of microbial population.

The structure of the program allows manual counting of microalgal cells captured in images obtained, using a simple toolbar with the commands such as Save, Open File, Number of Counts, and Reset Counting. The counting recorded for each image of microalgae can be recorded as a blue dot, and the number of elements count is gradually recorded. When counting finishes, the image is saved with the same name of the original file, with the number of elements represented in brackets. Images are count in the same orientation as the direct count, and the final cell density is calculated according to Table 2. Growth curves of the microalgae can be adjusted through linear regression, indicating that the plotted data could be fit to a straight line corresponding to the smaller distance between every experimental result and the straight line, with the purpose of reducing the sum of quadratic averages of the points. The Hypothesis test was also conducted, calculating Snedecor's *F*-Distribution of and Student *t*-test, based on the following hypothesis:

(1) y=α+βx,

where *x* and *y* correspond to the experimental values represented in the growth curves; *α* is the interception constant of the line with the *y* axis and *β* the slope of the curve.

**First.**

Hypothesis *H*_0_: tested through *F* distribution of Snedecor:

(2) $$\begin{array}{c} H_{0}={\left(s_{1}\right)}^{2}={\left(s_{2}\right)}^{2}=?,\\ F=\frac{s_{2}^{2}}{s_{1}^{2}}, \end{array}$$

where *F*: distribution; (*s*_1_)^2^ and (*s*_2_)^2^: variance of slope *β* of the lines.

**Second.**

Hypothesis  *H*_1_: tested through Student* t*-test:

(3) $$\begin{array}{cc} & H_{1}=\left(X_{1}\right)=\left(X_{2}\right)=?, \end{array}$$

(4) $$\begin{array}{cc} & t=\\ & \;\frac{X_{1}-X_{2}}{\sqrt{\left(\left(n_{1}-1\right)\cdot s_{1}^{2}+\left(n_{2}-1\right)\cdot s_{2}^{2}\right)/\left(n_{1}+n_{2}-2\right)}\cdot \sqrt{1/n_{1}+1/n_{2}}}, \end{array}\;$$

where  *X*_1_ and  *X*_2_ coefficients  *β* from the linear regression of the lines,  (*s*_1_)^2^ and  (*s*_2_)^2^: variance of the slopes of the lines, and  *n*_1_ and  *n*_2_: the number of data in the adjusted line.

In order to ensure the validity of using the software for the purpose of counting the cells, some comparative tests were performed: (a) a comparative study of the methodologies of direct counting using microscope and Fuchs-Rosenthal chamber with the software. Percent differences between the methods were then determined. The results, in these cases, were obtained using the *F* distribution of Snedecor and Student *t-*test; (b) a study of the reproducibility of countings using the results obtained through the software. Six images obtained were then used for these studies. Three random images obtained from each microalgae were selected. For each group of images, cells were counted 10 times, consecutively, reporting the average and standard deviation of the method.

## 3. Results and Discussion

### 3.1. Effect of pH on the Growth of the Microalgae

**Figure 2:**
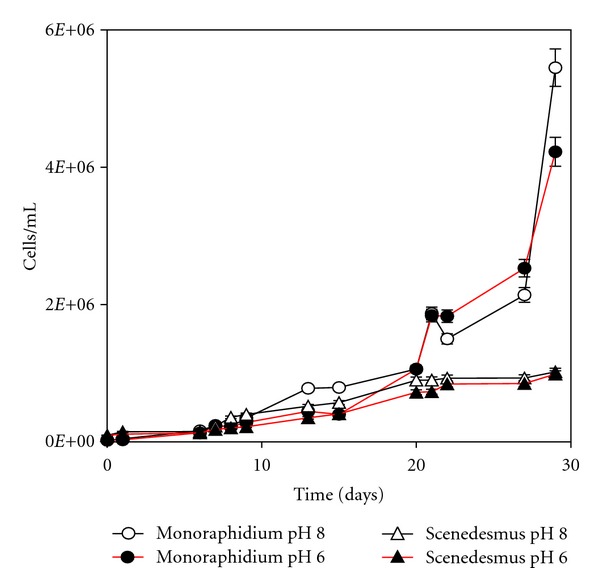
Growth curves of *Monoraphidium* and *Scenedesmus* cells, at pH 6.0 and 8.0 in ASM-1 culture medium.

Figure 2 represents the growth curves of both microalgae at pH 6.0 and 8.0.

It can be observed that the species *Monoraphidium* sp. presented similar growth, during almost 30 days, irrespective of the pH value. The growth became more pronounced from the middle of the period, reaching 5.45 × 10^6^ cells/mL at pH 8.0 and 4.23 × 10^6^ cells/mL at pH 6.0. These results indicate that the effect of pH was not very pronounced from 6.0 to 8.0. This is an important observation, as the speciation of thorium ions in solution is highly dependent on the pH value, and its ionic form is favored at lower pH values. Thus, pH 6.0 was selected for further studies.

It also shows the growth of the culture *Scenedesmus *sp. in ASM-1 medium, also in the absence of thorium in solution, at pH 6.0 and 8.0. It can be considered that the culture presented a similar growth, at both pH values, during 30 days. In the initial stages of the experiment, the microalgal population was equal to 9.35 × 10^4^ cells/mL, reaching 9.86 × 10^5^ cells/mL at pH 6.0 and 1.02 × 10^6^ cells/mL at pH 8.0, indicating that no substantial effect was observed due to pH change. As well, pH 6.0 was also selected for future tests with *Scenedesmus* cells.

### 3.2. Thorium Toxicity Tests: *Monoraphidium* sp. Contaminated with Thorium up to 100.0 mg/L

**Figure 3:**
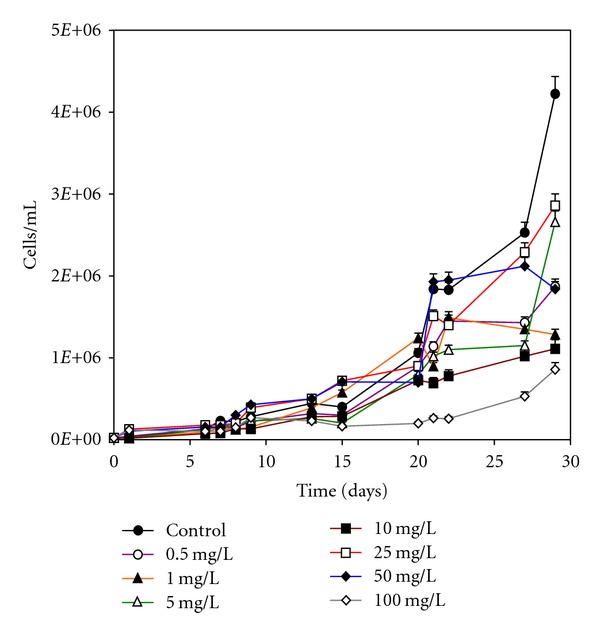
*Monoraphidium* sp. growth in the absence and presence of thorium up to 100.0 mg/L.

Figure 3 represents the direct count of microalgal populations, at pH 6.0, in absence and presence of thorium in the culture medium, up to 100 mg/L thorium. According to the results obtained through direct microscopic counting, the control experiment reached a final cell concentration equal to 4.23 × 10^6^ cells/mL. Cultures contaminated with thorium at 0.5, 1.0 and 5.0 mg/L grew accordingly, not indicating significant changes for the range of concentrations tested. However, *Monoraphidium* sp. culture contaminated with 10.0 mg/L thorium grew up to 1.11 × 10^6^ cells/mL, indicating some inhibition of growth due to the presence of the radionuclide in solution.

It can be observed that the growth of the cells start to differentiate from the 9th day. Particularly, the 10.0 mg/L thorium solution seemed to face the higher cell productivity, indicating that thorium, apparently, stimulated growth. However, it is well known that this radionuclide has no biological function. On the other side, it can be considered that some component in the culture medium could have reacted with thorium, decreasing its availability in solution, contributing for this stimulation. This hypothesis can be corroborated by the fact that nitrate, from the addition of thorium nitrate, could have stimulated growth, up to this concentration. For thorium concentrations equal to 10.0 and 25.0 mg/L, the toxic effect of thorium on the growth of *Monoraphidium* sp. cells was not so pronounced, as expected.

A detailed search in the published literature indicated that no documents are available for comparison including this radionuclide and its interaction with this type of biomass. Thus, the comparison of the present results with other authors becomes quite jeopardized. On the other side, the novelty associated with this research stimulates the continuation of this investigation for a possible new bioindicator.

### 3.3. Thorium Toxicity Tests: *Scenedesmus* sp. Contaminated with Thorium up to 10.0 mg/L

**Figure 4:**
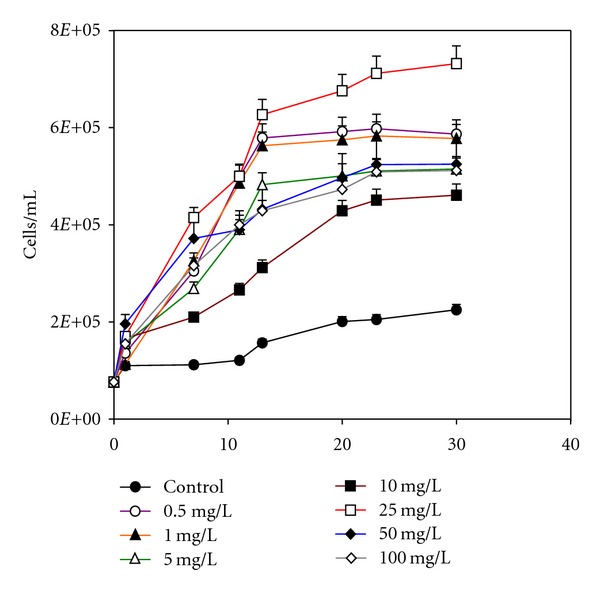
*Scenedesmus* sp. growth in the absence and presence of thorium up to 100.0 mg/L.

Figure 4 shows cell counts during growth of *Scenedesmus* sp. cells in absence and presence of thorium, through microscopic direct count.

It can be see that cultures contaminated with thorium presented a more pronounced growth in comparison with the control. A first approach of this fact suggests that the presence of the nitrate ion from the stock thorium solution could have been used by the cells as a nutrient source. Beyond this fact, it is possible that EDTA present in ASM-1 culture medium acted as a complexing agent for thorium, preventing its interaction with the microalgae, as already previously suggested.

The control experiment presented growth of the cells 61% smaller than the culture contaminated with 0.5 mg/L thorium, the higher growth. Even smaller than the culture contaminated with 0.5 mg/L thorium, the growth of cells in presence of 10.0 mg/L thorium was also pronounced, reaching a maximum value of 4.61 × 10^5^ cells/mL. *Scenedesmus* sp. cells contaminated with 1.0 mg/L thorium was equivalent to the medium containing 0.5 mg/L thorium. The remaining media, containing thorium at 5.0 and 10.0 mg/L, presented intermediate growth patterns.

At the same cultivation conditions, the microalgae *Monoraphidium* sp. was inhibited by thorium, decreasing its concentration in comparison to the control. This indicates the increased resistance of *Scenedesmus* sp. cells to the radionuclide.

Differently from the previous experiments, the growth of *Scenedesmus* sp. cells in thorium-contaminated media from 10.0 to 100.0 mg/L presented a distinct behavior, as compared to *Monoraphidium* sp. The control culture presented a higher growth in comparison to the thorium-contaminated media. It can also be observed that increasing thorium concentrations in solution contributed for a decrease in cell concentration. Cultures contaminated with 10.0 and 25.0 mg/L thorium solution could grow only 16% and 26% less than the control, respectively. Cultures containing 50.0 and 100.0 mg/L thorium were markedly affected by the presence of the radionuclide, presenting smaller cell productivities. In this case, the same behavior observed for *Monoraphidium* sp. cells was here observed. This distinct behavior between both microalgae may be associated to the different action and the distinct amount of nitrate reductase in the species, constituting the initial step in nitrate reduction.

Amado et al. [12] observed that microalgae from these genera were able to bioaccumulate copper on their surface structures, with a marked decrease in cell productivity, as a consequence. *Monoraphidium* sp. cells were tested, at the same experimental conditions used in the present work, and it could be observed that copper concentrations around 0.7 mg/L caused a significant inhibition of cell growth. Higher copper concentrations corroborated this behavior, being the 4.5 mg/L copper concentration totally inhibitory to cell growth of this species. *Scenedesmus* sp. cells were a little more resistant to the presence of copper in solution but analogously were completely inhibited by the presence of copper at 4.5 mg/L.

Marini [11] also studied the toxicity of ionic copper on the growth of photoautotrophic cells from the genus *Anabaena* and *Oscillatoria*. The author observed that, in both cases, the toxicity of copper was already pronounced in the concentration of 1.0 mg/L, indicating that these cells were extremely sensitive to the presence of ionic copper in solution.

In the published literature, no other publications were found, correlating radionuclides interacting with microbial cultures tested in the present study. Thus, the comparison of the present work with the published literature was not possible, making these results very particular. However, the literature reports the use of seaweeds for the biosorption or uranium and thorium [4, 13–15]. In those papers, seaweeds were used as inactivated biomass, under batch and continuous conditions, with the purpose of treating effluents containing radionuclides. The purpose of those works was to concentrate metallic elements on the surface of the seaweeds aiming at a controlled storage of the radionuclides. In those cases, no metabolic reactions were involved in the process, due to the use of inactivated biomass, thus preventing quantitative comparisons with the present work. 

### 3.4. Comparison between Direct Count and Software Count of the Cells

Table 3 compares microscopic direct count and software count, for the growth of *Monoraphidium* sp. and *Scenedesmus *sp. in ASM-1 medium at pH 6.0, in the absence of thorium, and contaminated with the radionuclide up to 100.0 mg/L thorium.

In general, the differences between both counts were not statistically significant. In this case, the software proved to be an important tool for the corroboration of results found, whose records can be kept for future studies. Additionally, results are obtained faster than direct count in the microscope.

Comparing *Monoraphidium* sp. counts using both techniques indicate that when the software is used, absolute cells counts obtained were smaller, although not statistically distinct. An opposite behavior was observed when *Scenedesmus* cells were counted, although not statistically distinct, as well. This behavior gives an indication that morphologic characteristics of the cells can have affected cell counts, when using the software, as cells densities were similar for both species.

### 3.5. Reproducibility of Counts Using the Software

The reproducibility test, both for *Monoraphidium* sp. and *Scenedesmus* sp. cells, showed that after ten consecutive counts, the standard deviation reached a maximum value of 0.63 for high densities of the cell cultures of *Monoraphidium* sp., confirming that the method is reliable and reproducible, markedly contributing for an improvement in the estimates of cell count. This statement was also confirmed for high, medium, and low cell densities. The same results were obtained for *Scenedesmus* sp. cells, as well. In this case, however, the maximum standard deviation reached the maximum value of 2.35 for high cell densities.

### 3.6. Images from the Culture of *Monoraphidium* sp

**Figure 5:**
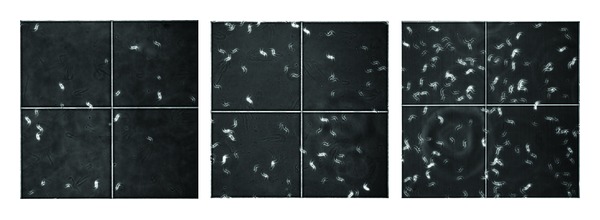
*Monoraphidium *sp. culture in absence of ^232^Th (left); in presence of 0.5 mg/L ^232^Th (center); in presence of 10.0 mg/L ^232^Th (right) (20-fold increase).

Figure 5 (left) represents the image of a culture of *Monoraphidium* sp. in ASM-1 medium in the absence of thorium, at pH 8.0, where the low cell density can be observed. The central figure represents the same cell culture, contaminated with 0.5 mg/L thorium in the sixth day of growth. In the right figure, it can be observed the same culture contaminated with 10.0 mg/L, in the same experimental conditions. In these cases, from the 1st to the 3rd images represented, it can be observed the formation of small aggregates that difficult counting based on direct microscope counting.

### 3.7. Images from the Culture of *Scenedesmus* sp

**Figure 6:**
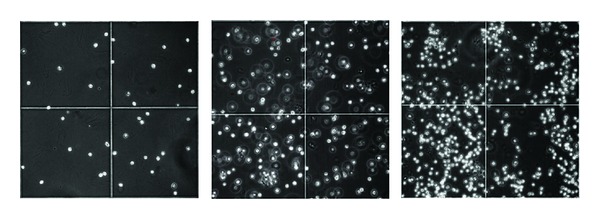
*Scenedesmus *sp. culture in absence of ^232^Th at pH 6.0 (left); in absence of ^232^Th at the 7th day of growth (center); in the 23rd day of growth (right) (20-fold increase).

Figure 6 (left) represents an image of *Scenedesmus *sp. culture, in ASM-1 medium, at pH 6.0 in absence of thorium.

It can be seen the low cell density in the medium, widely spread all over the image. On the 7th day of culture of the cells, the aggregate cells become gradually difficult to count through conventional methods (Figure 6, center). A detailed microscopic description of the image indicates the formation of aggregates in more than one plane, markedly jeopardizing the count. As well, the lack of a suitable focus for observation in different planes also contributes for an incorrect estimation of cell density. On the right side of the image (Figure 6, right), the same culture is presented in the 23rd day of growth. Again, the problem associated with the lack of focus and increased formation of cell aggregates is here more pronounced.

## 4. Conclusions

The different values of pH tested, 6.0 and 8.0, did not affect significantly cell growth for both *Monoraphidium* sp. and *Scenedesmus* sp. cultures, using ASM-1 medium.

The culture of *Monoraphidium *sp. was resistant to the presence of thorium in solution, in comparison to control tests. Just the concentrated 50.0 and 100.0 mg/L thorium solution inhibited cell growth, decreasing its concentration tenfold.

When *Scenedesmus* sp. cells were used, cell growth was stimulated by increasing thorium concentrations, probably to an increase of nitrate in the medium. Just high concentrations, such as 50.0 and 100.0 mg/L thorium, changed cell growth. A possible explanation for this fact can also be attributed to the presence of EDTA in solution, a complexing agent that could have removed thorium from solution, preventing its toxicity to cells.

The software used for counting the cells was very efficient for this purpose, preventing mistakes in the estimation of cell density, mainly for high cell populations, presenting good reproducibility, and corroborating the importance of this technique.

The use of *Monoraphidium* sp. and *Scenedesmus* sp. cells opens the possibility for future investigations on the use of these cultures as bioindicators of thorium contamination due to their particular behavior in presence of this radionuclide.

This paper contributed with a novelty related to the use of metabolically driven processes to recover a radioactive element from a liquid solution, under controlled conditions. As well the possibility of using software to monitor the effect of chemicals on the growth of cells also confirms that it is possible to open this field for investigations using other types of microbial cells and radioactive elements.

## Figures and Tables

**Table 1:** Commercial cell-counting chambers [9].

Counting chamber	Cell size (*μ*m)	Culture density (cells/mL)
Sedgwick-Rafter	50–500	30–10^4^
Palmer-Maloney	5–150	10^2^–10^5^
Speirs-Levy	5–75	10^4^–10^6^
Fuchs-Rosenthal	5–75	10^4^–10^6^
Neubauer	2–30	10^4^–10^7^
Petroff-Hausser	<1–5	10^6^–10^8^

**Table 2:** Conversion table for counted cells to cells density [11].

Area considered	Cell density (cells/mL)
1/16 de A	N × 8000 × 10 × f
1/8 de A	N × 4000 × 10 × f
1/4 de A (4a)	N × 2000 × 10 × f
1/2 de A (8a)	N × 1000 × 10 × f
1A (16a)	N × 500 × 10 × f
2A (32a)	N × 250 × 10 × f
4A (64a)	N × 125 × 10 × f
8A (128a)	N × 62.50 × 10 × f
16A (256a)	N × 31.25 × 10 × f

A: large squares; a: small squares; N: counted cells; f: dilution factor.

**Table 3:** Comparison between direct cell counts and software counts.

Days of cultivation	*Monoraphidium/Scenedesmus *(cells/mL)	
Blank (no Th addition)	Direct count	Software count
0	2.27*E* + 04/7.65*E* + 04	2.06*E* + 04/7.20*E* + 04
10	2.81*E* + 05/1.57*E* + 05	2.66*E* + 05/1.99*E* + 05
30	4.23*E* + 06/2.25*E* + 05	1.78*E* + 06/3.76*E* + 05

0.5 ppm Th		
10	2.21*E* + 05/5.79*E* + 05	1.57*E* + 05/4.27*E* + 05
30	1.87*E* + 06/5.87*E* + 05	1.19*E* + 06/6.63*E* + 05

1.0 ppm Th		
10	1.54*E* + 05/5.63*E* + 05	1.77*E* + 05/4.43*E* + 05
30	1.29*E* + 06/5.78*E* + 05	9.02*E* + 05/6.60*E* + 05

5.0 ppm Th		
10	2.32*E* + 05/4.83*E* + 05	1.73*E* + 05/4.20*E* + 05
30	2.66*E* + 06/5.15*E* + 05	1.10*E* + 06/6.30*E* + 05

10.0 ppm Th		
10	1.32*E* + 05/3.12*E* + 05	1.22*E* + 05/4.47*E* + 05
30	1.11*E* + 06/4.61*E* + 05	6.85*E* + 05/6.70*E* + 05

25.0 ppm Th		
10	3.91*E* + 05/6.27*E* + 05	6.85*E* + 05/9.98*E* + 05
30	2.86*E* + 06/7.32*E* + 05	2.58*E* + 06/1.21*E* + 06

50.0 ppm Th		
10	4.27*E* + 05/4.32*E* + 05	7.20*E* + 05/5.12*E* + 05
30	2.12*E* + 06/5.25*E* + 05	2.27*E* + 06/4.70*E* + 05

100.0 ppm Th		
10	2.70*E* + 05/4.29*E* + 05	1.68*E* + 05/3.59*E* + 05
30	8.57*E* + 05/5.12*E* + 05	6.60*E* + 05/4.12*E* + 05
